# IGF-II induced by hepatitis B virus X protein regulates EMT via SUMO mediated loss of E-cadherin in mice

**DOI:** 10.18632/oncotarget.10922

**Published:** 2016-07-29

**Authors:** Hye-Lin Ha, Taeho Kwon, In Seon Bak, Raymond L. Erikson, Bo Yeon Kim, Dae-Yeul Yu

**Affiliations:** ^1^ Disease Model Research Laboratory, Genome Editing Research Center, Korea Research Institute of Bioscience and Biotechnology (KRIBB), Daejeon, Republic of Korea; ^2^ Department of Functional Genomics, University of Science and Technology, Yuseong-gu, Daejeon, Republic of Korea; ^3^ Department of Molecular & Cellular Biology, Harvard University, 16 Divinity Avenue, Cambridge, MA, USA; ^4^ Anticancer Agents Research Center, Korea Research Institute of Bioscience and Biotechnology (KRIBB), Ochang, Cheongwon, Republic of Korea; ^5^ Present address: Immune Activation Section, Laboratory of Molecular Immunology, National Institute of Allergy and Infectious Diseases, National Institutes of Health, Bethesda, MD, USA

**Keywords:** hepatocellular carcinoma, hepatitis B virus X protein, IGF-II, epithelial-mesenchymal transition, SUMOylation

## Abstract

Hepatocellular carcinoma (HCC) is one of the most common cancers and a leading cause of cancer mortality. Prognosis of this disease largely depends on its stage. An Enlarged liver, due to dysplasia, may be a critical point in the multi-step progression to HCC. The mechanism underlying hepatomegaly in human and mouse models are poorly understood. We previously reported we observed enlarged liver in hepatitis B virus X protein (HBx) expressing mice (HBx mice). Here we identify the critical role of HBx induced IGF-II in hepatomegaly in mice and abnormal cell growth in human hepatoma cells. We found that HBx induced IGF-II is essential to induce epithelial-mesenchymal transition (EMT) through loss of E-cadherin. In mouse liver, loss of E-cadherin was mediated by post-translational regulation, at least in part, by protease and SUMOylation not by transcriptional regulation. In contrast, in hepatoma cell line (HepG2 cells) Akt signal pathway controls the mRNA expression level of EMT-related transcription factors, especially Twist, in addition to post- translational modification through SUMOylation. Thus, IGF-II-mediated loss of E-cadherin is central in developing hepatomegaly in mice and abnormal cell growth in the hepatoma cell line. HBx induced IGF-II represents a potential biomarker, which is also a therapeutic target in HCC.

## INTRODUCTION

Hepatocellular carcinoma (HCC) is the fifth most common type of cancer worldwide and the second leading cause of cancer mortality [1]. Although the prognosis for patients with HCC is generally poor (about 15%), the 5-year survival rate is much improved (up to 70%) if patients are diagnosed at an early stage [2]. The finding that HCC appears with chronic hepatitis and/or cirrhosis in regions of liver-cell dysplasia or adenomatous hyperplasia [3], suggests that tumors develop from a series of histologically identifiable lesions that precede tumor development. Definitive evidence indicates that molecular pathogenesis causes HCC, but focused mainly on late-stage carcinogenesis not early stage. Thus it's interesting to investigate molecular pathogenesis of early stage of carcinogenesis.

Among other risk factors, chronic hepatitis B virus (HBV) infection plays a central role in the etiology of HCC [4]. Among the four proteins encoded by the HBV genome, HBV X (HBx) is a multifunctional regulatory protein closely linked to HCC, but its role in tumor growth is incompletely understood. Previously others and we showed that HBx induces liver cancer in transgenic mice (HBx mice) [5–7]. HBx does not bind directly to DNA, but affects transcriptional activation through interaction with nuclear transcription factors and by cytoplasmic modulation of signal transduction pathways [8]. HBx also activates growth-stimulatory pathways. HBx up-regulates the expression of insulin-like growth factor-II (IGF-II) in premalignant proliferative nodules [9] and type I IGF receptor (IGFIR) in hepatoma cell lines [10], implying that HBx may set up an autocrine loop [11] that enhances cell growth independent of other serum growth factors.

Insulin-like growth factors (IGFs) are essential for normal growth and development [12]. IGFs exert biological actions primarily by binding and activation of the IGFIR. Dysregulation of the IGF system aligns with many developmental disorders and pathological conditions. Cancer is one disease in which the IGF system is dysregulated. In particular, IGF-II, an autocrine growth factor, is highly expressed in many tumors and tumor cell lines [13]. At the cellular level, IGF-II induces a variety of cellular responses, including cell proliferation, differentiation, migration, and survival. In particular, IGF-II has a role in embryonic and postnatal-growth [14] of the liver. IGF-II overexpression resulting from loss of imprinting often aligns with somatic overgrowth [15].

Epithelial-mesenchymal transition (EMT) plays an important role in embryogenesis and carcinogenesis [16]. EMT occurs frequently during development, at different times and in distinct organs [17], including the liver [18]. EMT may also be an important step in leading to invasion and metastasis [19]. At the molecular level, loss of epithelial cell markers, including the cell-adhesion protein, E-cadherin [20] and acquisition of mesenchymal markers, such as vimentin [21] characterize EMT. E-cadherin is an essential component of adherens and acts as a tight junction in polarized epithelial cells [22]. In most cases during the EMT, E-cadherin expression is downregulated at the transcriptional level. The Snail, Slug, ZEB1 and Twist are important transcriptional repressors of the E-cadherin gene and actively repress its expression [23, 24]. IGF-II is able to induce an EMT, concomitant with the loss of the E-cadherin function. Curiously, following IGF-II stimulation, E-cadherin internalizes and degrades by an unknown mechanism [25].

Posttranslational modifications of proteins play a critical role in most cellular events. Ubiquitination (Ub) is well-known protein degradation by posttranslational modification. More recently, a family of polypeptides distantly related to Ub called small ubiquitin-like modifiers (SUMOs) has emerged as a second influential modifier [26]. Recent studies suggested the possible involvement of SUMO in pathological conditions such as cancer, in addition to its regular function in normal cells [27]. Mammals have three SUMO paralogues (SUMO1, SUMO2 and SUMO3) [28]. Similar to ubiquitin, SUMOs can be attached to cellular target proteins. SUMO can act as a signal to recruit E3 ubiquitin ligases, which lead to the ubiquitylation and degradation of the modified protein mechanism [29, 30].

Here we describe mechanisms by which HBx contributes to specific HCC pathologies. We demonstrated that HBx induced IGF-II critically alters cell growth. And it's due to disruption of the cell-cell interaction via SUMO-mediated loss of E-cadherin. These findings suggest that IGF-II acts as a potential diagnosis marker and therapeutic target in HCC.

## RESULTS

### HBx induces abnormal cell growth in mouse livers and HepG2 cells

**Figure 1:**
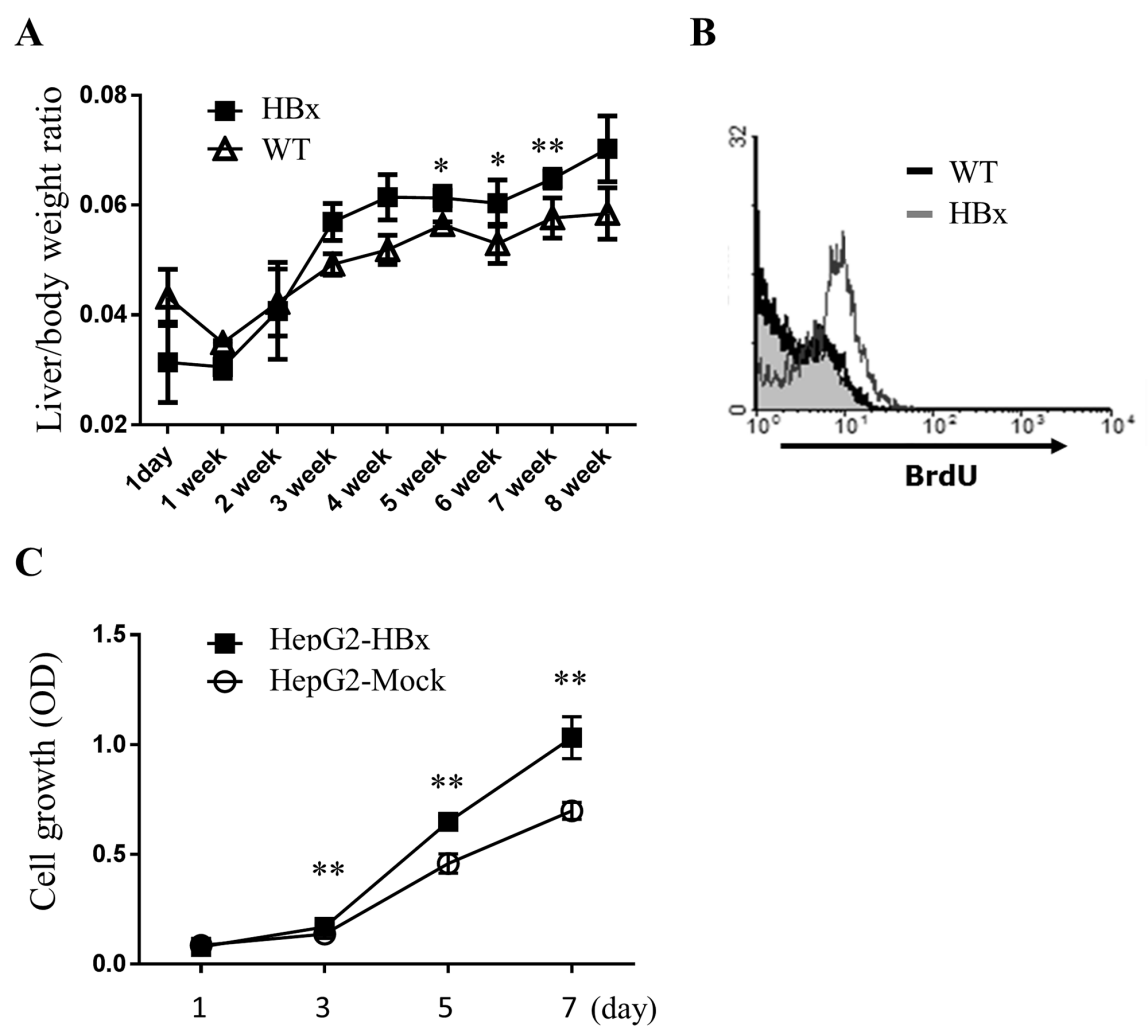
HBx increases cell growth in livers of mice and HepG2 cells **A.** The ratio of liver weight per body weight of WT and HBx mice was quantified. Mean ± *SD*; * P< 0.05, ** P< 0.01, n=5 **B.** BrdU incorporation of primary hepatocytes was measured by flow cytometric analysis. **C.** Cell-proliferation assays were performed with HepG2-Mock and HepG2-HBx cells. Mean ± *SD*; ** P< 0.01, *n*=9 per group.

Previous studies suggested that liver enlargement is a clinical sign of some underlying disease including HCC [31]. We observed that HBx expression mice showed hepatomegaly even before they had pathological regions. In wild-type (WT) mice, the liver body weight ratio converged around 5 or 6 weeks after birth, but in HBx mice, the live body weight ratio continually increased (Figure 1A). No significant difference in cell size emerged with flow cytometric analysis of hepatocytes (data not shown). However, hepatocytes from HBx mice showed higher BrdU incorporation compared to WT mice (Figure 1B), suggesting that hepatomegaly in HBx mice was caused by increased cell numbers through increased proliferation of hepatocytes, not through enlarged cell size.

HBx expressing HepG2 cells (HepG2-HBx) (Supplementary Figure S1B) also showed a higher rate of cell growth. The growth rate of HepG2-HBx cells approximately doubled compared with control cells (HepG2-Mock) (Figure 1C). In sum, expression of HBx markedly promotes cell growth in mouse livers and HepG2 cells.

### HBx induced IGF-II plays a key role in abnormal cell growth in mouse livers and HepG2 cells

**Figure 2:**
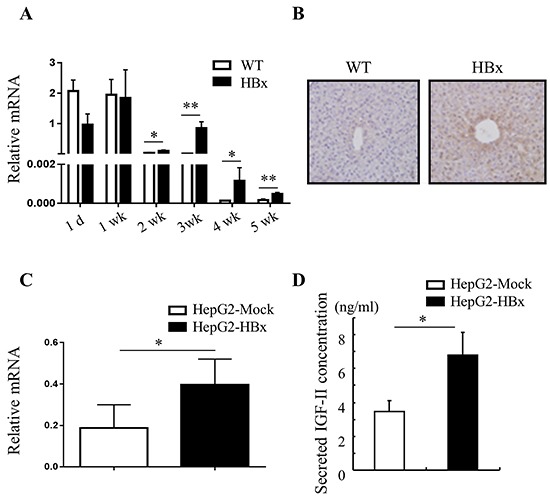
HBx induces IGF-II mRNA and protein expression **A.** and **C.** Relative mRNA expression of IGF-II in WT and HBx mouse livers (A) Mean ± *SD*; * P< 0.05, ** P< 0.01, n=3, HepG2-Mock and HepG2-HBx cells (C) Mean + *SD*; * P< 0.05, n=5-8. **B.** Immunohistochemistry images of 3-week-old WT and HBx-mouse livers stained with IGF-II. Original magnifications 40X. Data are representative of 3-5 mice per group. **D.** Secreted IGF-II protein levels from HepG2-Mock and HBx cells were measured. Mean ± *SD*; * P< 0.05, n=6.

To elucidate the mechanisms by which HBx mediated signaling contributes to overgrowth, we examined the mRNA expression of several growth factors and receptors relevant to hepatomegaly. The gene expression levels of growth factors such as HGF, EGF, IGF-I, IGF-signaling receptors IGF-1R, IGF-IIR and IRS-1 were not different between HBx mice and WT mice liver (Supplementary Figure S2). But stark contrast arose in the expression pattern of fetal liver growth factor, IGF-II, which has been proposed to correlate with hepatomegaly [32], in HBx and WT mice liver. IGF-II is predominantly active in fetal liver under physiological conditions and normally its expression is strictly down-regulated shortly after birth by imprinting mechanism [33]. After birth (1-d to 1-wk) WT and HBx mice showed high expression level of IGF-II. In 2 weeks old mice (WT and HBx), IGF-II expression started to decrease. Down regulation of IGF-II was greatly attenuated, but not abolished in HBx livers (Figure 2A and 2B). No correlation emerged with the IGF-II specific transcription factor Gli (data not shown) but it was correlated with expression of HBx (Supplementary Figure S1A). Also no significant differences emerged in expression of other imprinted genes such as Zrsr1, GNAS and MEST between WT and HBx mice (Supplementary Figure S2). These findings suggest that over expression of IGF-II in HBx mouse is not due to loss of imprinting.

**Figure 3:**
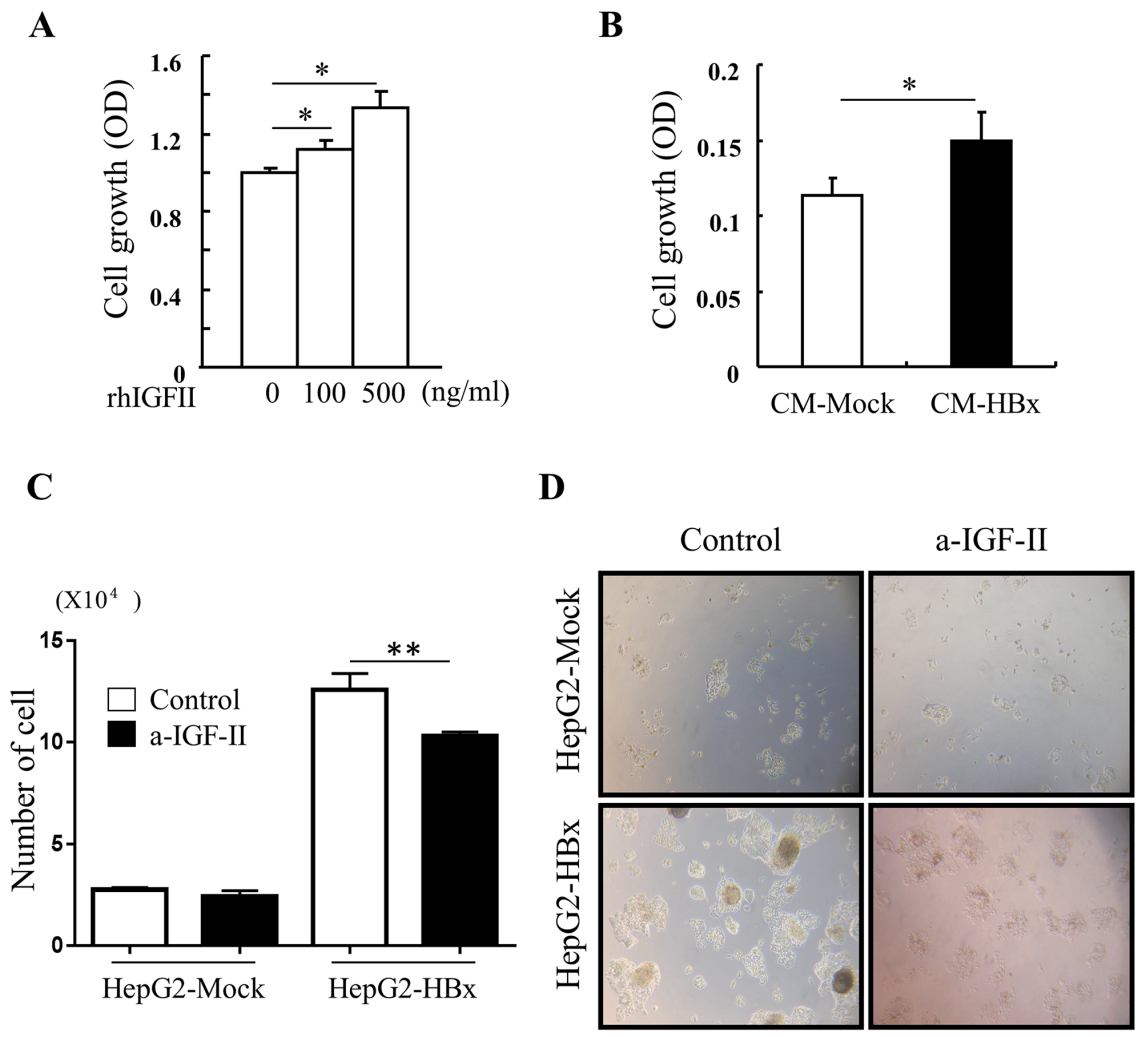
IGF-II induces cell growth and IGF-II neutralization reverses abnormal cell growth A. cell growth was measured in serum starved HepG2-Mock cells treated with rhIGF-II (0-500 ng/ml) for 24 h Mean ± *SD*; * P< 0.05, n=6. **B.** Cell growth was measured in HepG2-Mock cells treated with conditioned medium from HepG2-Mock (CM-Mock), and HBx (CM-HBx). Mean ± *SD*; * *P*< 0.05, n=6. **C.** Cell numbers were quantified in HepG2-Mock and HBx cells with or without anti-IGF-II. Mean ± *SD*; ** P< 0.01, n=4. **D.** Multilayer growth of HepG2-HBx cells were reversed to monolayer growth with anti-IGF-II.

A high level of IGF-II expression defined HepG2 cell lines compared with other cancer cell lines [34]. Nevertheless, over expression of HBx in HepG2 cells led to significant increases in expression of IGF-II mRNA and production of secreted IGF-II protein than HepG2-Mock cells (Figure 2C and 2D). As expected, HepG2-Mock cells treated with rhIGF-II (100 or 500ng/ml) or a supernatant (conditioned media: CM) from HepG2-HBx proficiently increased growth rate (Figure 3A and 3B) and also altered their morphology to be similar to HepG2-HBx cells (data not shown). We next established that IGF-II induced by HBx promotes cell growth and change cell morphology, because anti-IGF-II antibodies abolished this effect. Scavenging IGF-II led to 20% inhibition of cell growth (Figure 3C) and significant change in cell morphology in HepG2-HBx cells, multi-layer to mono-layer growth similar to HepG2-Mock cells (Figure 3D).

Taken together, these *in vivo* and *in vitro* data indicate HBx induced IGF-II causes abnormal cell growth, which can be reversed by scavenging overexpressed IGF-II.

### IGF-II induces epithelial-mesenchymal transition (EMT) through downregulation of E-cadherin

Loss of cell-to-cell interaction can cause abnormal cell growth [35]. E-cadherin and β-catenin are expressed abundantly in the adult liver, and localize at cell–cell junctions. But during EMT, epithelial markers, E-cadherin and catenins, are downregulated [35].

**Figure 4:**
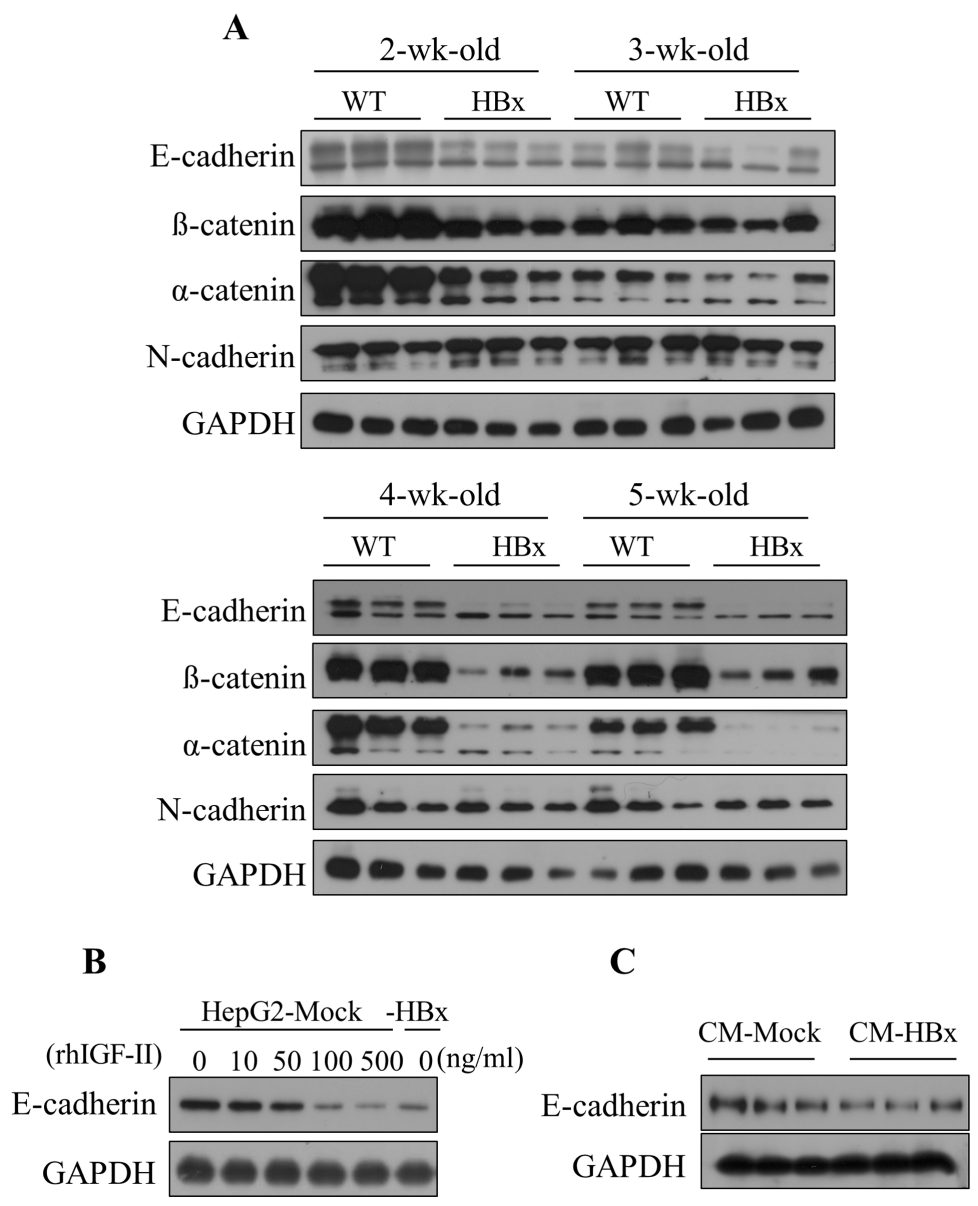
HBx induced IGF-II downregulates the expression level of E-cadherin in mouse livers and HepG2 cells **A.** The expression level of Epithelial markers (E-cadherin, α and β-catenin) and mesenchymal marker (N-cadherin) in 2 to 5 weeks of WT and HBx mouse livers. **B.** The expression level of E-cadherin in the serum-starved HepG2-Mock cells treated with rhIGF-II (0-500 ng/ml) for 24 hours. **C.** The expression level of E-cadherin in the HepG2-Mock cells treated with a HepG2-HBx-conditioned medium (CM-HBx) and a HepG2-Mock-conditioned medium (CM-Mock). GAPDH served as the loading control.

To investigate abnormal cell growth by HBx induced IGF-II effects, we first determined the mRNA expression levels of cell-cell junction molecules. From day 1 to 1 week WT and HBx mice showed high expression levels of E-cadherin. Surprisingly, after 2 weeks levels of E-cadherin in livers of HBx mice started to decrease. After 4 weeks the full length of E-cadherin has almost disappeared in HBx livers. In contrast, WT mice continued to maintain high expression of E-cadherin (Figure 4A). We found that expression levels of IGF-II and E-cadherin inversely related to each other. Consistent with these results, HBx livers also showed decreased levels of the epithelial markers, α and β-catenin, but no significant difference emerged in the expression level of the mesenchymal marker, N-cadherin. Consistent with the diminished E-cadherin in the liver of HBx mice, E-cadherin protein levels greatly diminished in HepG2-HBx compared to HepG2-Mock cells. In control cells, E-cadherin protein levels decreased after treatment with rhIGF-II in a dose-dependent manner (Figure 4B). In addition, treatment with the HBx-conditioned medium decreased E-cadherin levels in HepG2-Mock cells (Figure 4C). These findings indicate that over expression of IGF-II causes EMT through downregulation of E-cadherin in HBx mice and HepG2-HBx cells. Cells that lost cell-cell interactions led to abnormal growth.

### Loss of epithelial markers dose not align with EMT-related transcription signaling pathways in HBx mice

**Figure 5:**
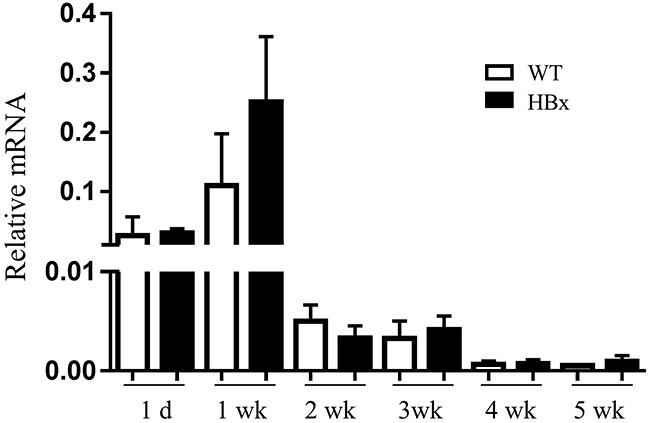
Relative mRNA expression for E-cadherin in the WT and HBx mouse livers from 1 day to 5 weeks Mean ± *SD*; *n*=9.

Src [36], Raf-1/MEK/ERK [37, 38], JNK [39], p38 [40] and STAT3 [41] pathways link with downstream of IGFIR activation and EMT. Activation of those pathways could increase EMT-inducing transcription factors such as Snail, Slug, ZEB1, and Twist which led to decreases in expression of epithelial markers, especially E-cadherin, and increases in expression of mesenchymal markers. Despite the large decrease in E-cadherin level, EMT related pathways (Src, MAPKs, STAT3 and NF-kB) did not activate in HBx mouse livers (Supplementary Figure S3). Furthermore, no significant differences arose in mRNA expression for EMT-inducing transcription factors (Supplementary Figure S4). To evaluate the loss of E-cadherin in HBx mice, we measured the mRNA expression level of E-cadherin. Unlike the protein level, no significant difference arose in mRNA expression level between WT and HBx mice. In contrast, the mRNA level of E-cadherin in HBx mice remained higher than in WT mice for an extended time (Figure 5). These findings suggest that loss of E-cadherin in HBx mice is not due to transcriptional regulation.

### In HepG2 cells IGF-II induces EMT regulated by Akt pathways

**Figure 6:**
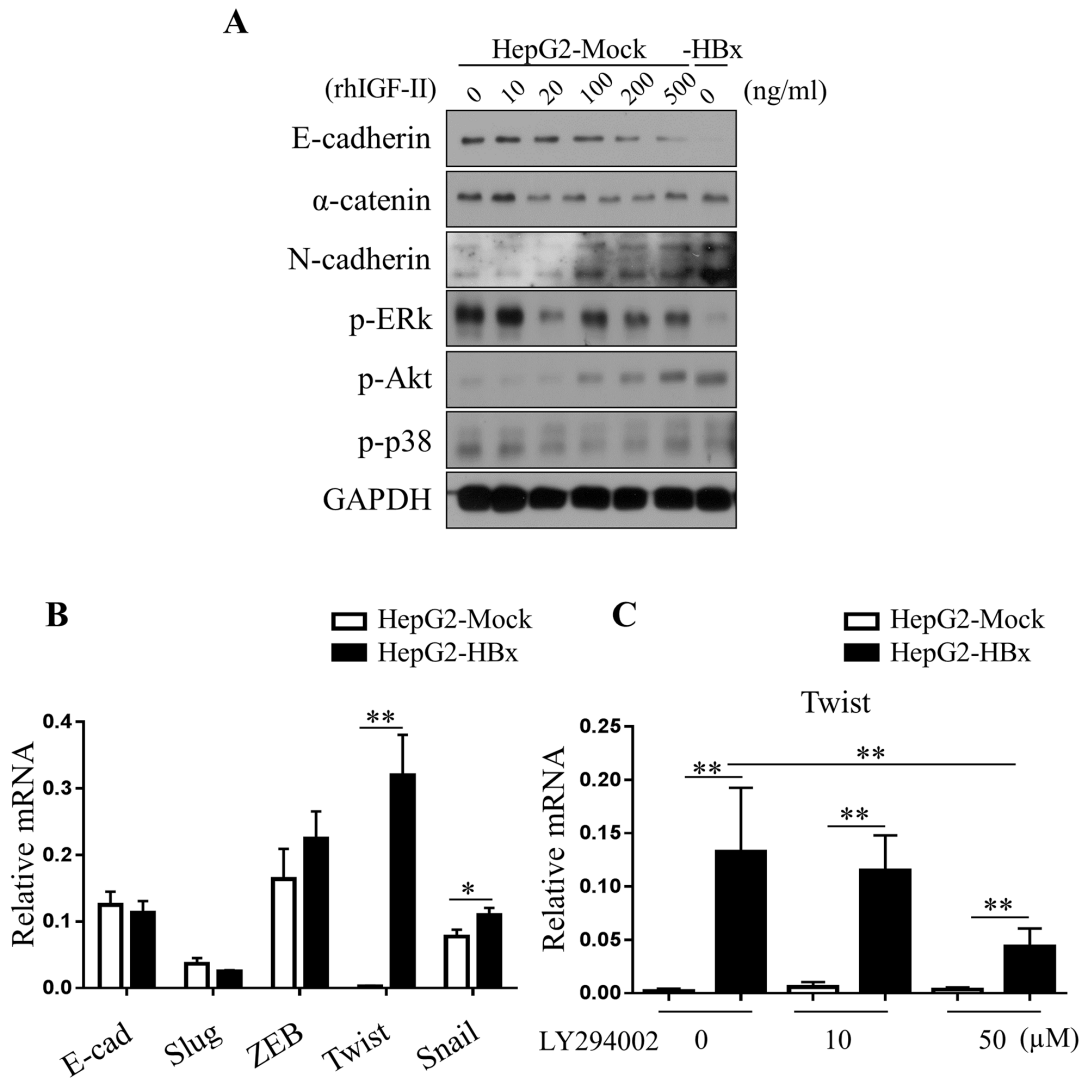
Loss of E-cadherin in HepG2 cells are dependent on Akt pathways **A.** Activation of Akt pathways (p-Akt) were observed in the serum starved HepG2-Mock and HBx cells treated with rhIGF-II (0-500 ng/ml). **B.** Relative mRNA expression levels of EMT-inducing transcription factors in HepG2-Mock and HBx cells were measured. Mean ± *SD*; * P< 0.05, ** P< 0.01, n= 6. **C.** Relative mRNA expression level of twist was measured in HepG2-Mock and HBx cells treated with an Akt inhibitor (LY294002, 0, 25 or 50μM) for 24 hours. Mean ± *SD*; * * P< 0.01, n= 9.

Among many pathways that could led EMT, we observed activation of the Akt pathway in HepG2-HBx cells compared to HepG2-Mock cells. Activation of Akt signaling with rhIGF-II treatment resulted in HepG2-Mock cells in a dose-dependent manner. Unlike EMT in the mouse liver, HepG2-HBx cells showed an increased level of the mesenchymal marker N-cadherin compared to control cells in a dose-dependent manner. mRNA and Protein expression level of α-catenin (another epithelial marker) diminished in HepG2-HBx cells and rhIGF-II treated HepG2-Mock cells (Figure 6A). These data indicate that IGF-II-induced Akt activation aligns with EMT in HepG2 cells. Previous studies suggested that activated Akt signaling induces expression of EMT-inducing transcription factors such as Twist [42, 43]. Therefore, we analyzed the mRNA level of several EMT-inducing transcription factors- Snail, Slug, ZEB1 and Twist in HepG2-Mock and HepG2-HBx cells. mRNA expression levels of Snail, Slug, ZEB1 and Twist increased in HepG2-HBx cells compared to control cells. Among them Twist showed the most significant increase (Figure 6B). As expected, the mRNA expression level of Twist significantly decreased when treated with an Akt inhibitor in a dose-dependent manner in HepG2-HBx cells (Figure 6C). These findings identify, at least in part, a mechanism by which IGF-II activates Akt signaling into HepG2 cells, leading to loss of E-cadherin and inducing EMT.

### Posttranslational modification mediates Loss of E-cadherin

**Figure 7:**
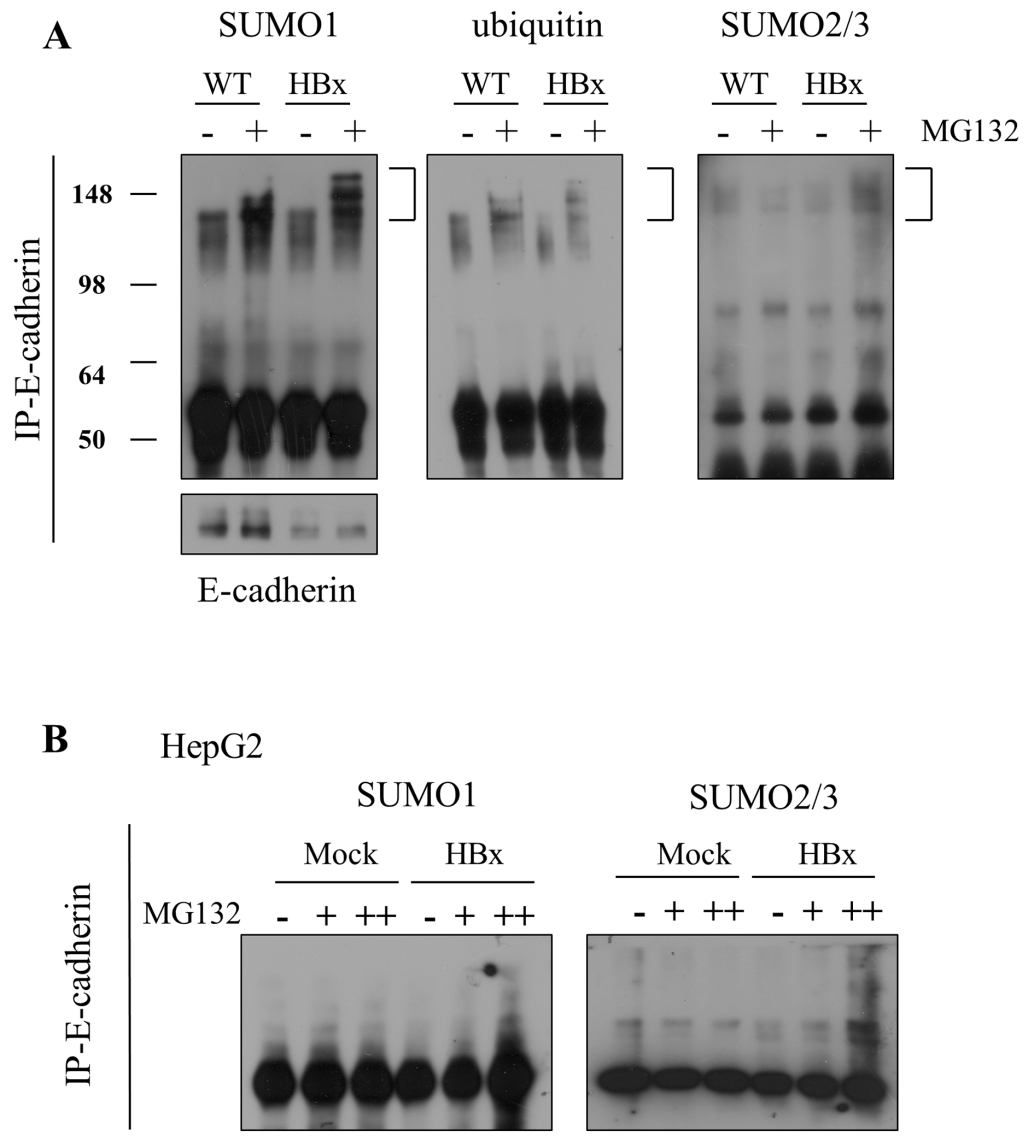
E-cadherin was degraded by SUMOylation in mouse livers and HepG2 cells **A.** SUMO1ylation, ubiquitination and SUMO2/3ylation of E-cadherin (left panel to right panel) were examined in 4-week-old WT and HBx primary hepatocytes, with/without treatment of 20μM of MG132. **B.** Analysis of SUMO1ylation and SUMO2/3ylation of E-cadherin in HepG2-Mock and HBx cells, with/without 20μM of MG132.

The data presented above demonstrate that HBx induced IGF-II promoted loss of E-cadherin and led to EMT *in vivo* and *in vitro*. *In vitro*, we were able to show translational regulation of E-cadherin through Akt-EMT-inducing transcription factors, especially the Twist pathway. But *in vivo* loss of E-cadherin was not due to translational regulation. Previous reports on posttranslational modification of E-cadherin showed protease MMP3 [44, 45], MMP7 [46] and ADAM10 [47] mediated cleavage and MDM2 [48] and newly founded c-cbl like ligase, hakai [49] mediated ubiquitination. Therefore we asked whether loss of E-cadherin in HBx mice might be due to post-translational regulation. First we checked the cleavage of E-cadherin by protease. HBx livers showed less E-cadherin protein, especially at 120 kDa full length. Through Western blot analysis, we were able to detect cleaved 40 kDa of E-cadherin in the protein lysate (Supplementary Figure S5). However, the amount of the cleaved form of E-cadherin was insufficient to explain the loss of E-cadherin in HBx mice. Another classical pathway for E-cadherin degradation is related to the E3 ubiquitin ligase MDM2. Surprisingly the protein level of MDM2 decreased in HBx mice like E-cadherin. However, no significant difference arose in levels of p53 protein, the key target of MDM2 (Supplementary Figure S6). Next, we tested another proteolysis mechanism, SUMOylation of E-cadherin. We first analyzed expression of SUMO proteins. We observed a diminished amount of the higher molecular size of SUMO1, which is the predominant form of SUMO1, in HBx livers. To detect if total SUMO1 decreases or if a molecular structure shifts in SUMO1, we analyzed the size of SUMO1 proteins. Interestingly, the larger sized SUMO1 decreased but the smaller sized SUMO1 increased (Supplementary Figure S7A). Compared with SUMO1 a large quantity of a free pool of SUMO2/3 emerged. However no significant difference arose in the form of SUMO2/3 itself in livers (Supplementary Figure S7B). We speculated that abnormally increased free SUMO1 caused E-cadherin degradation via SUMO1ylation. SUMO1ylation of E-cadherin significantly increased in HBx hepatocytes after treatment with a proteasome inhibitor MG132. Ubiquitination and SUMO2/3ylation of E-cadherin also slightly increased in HBx hepatocytes (Figure 7A). Through this data, we conclude that SUMO1ylation of E-cadherin is the dominant modification in E-cadhrin degradation.

In contrast, in HepG2 cells, no free form of SUMO1 emerged and we detected only the conjugated form of SUMO1; also, no difference arose between HepG2-Mock and HepG2-HBx cells (Supplementary Figure S7C). SUMO2/3 decreased in HepG2-HBx cells compared to HepG2-Mock cells. In the HepG2 cancer-cell line, we did not detect the over-90 kDa conjugated form of SUMO2/3 (Supplementary Figure S7D). As we expected, HepG2–HBx cells showed more specific SUMO2/3ylation than SUMO1ylation of E-cadherin (Figure 7B).

In aggregate, these data indicate that loss of E-cadherin via SUMOylation plays an important role in HBx induced EMT but, surprisingly, is tightly regulated by different pathways of SUMO modification.

## DISCUSSION

HCC is one of the most frequent tumor types worldwide, with more than estimated 700,000 new cases annually [50]. Many findings implicate HBx as a major risk factor for HCC. To date most studies focused on HBx and HCC. However, little is known about how HBx contributes to early events that precede pathogenesis of hepatocellular dysplasia, transformation, or HCC. For an effective cure for liver disease, identifying an early diagnostic and treatment target is important and needed. Here we investigated whether HBx is critical in the early and late development of HCC and what its contributions and underlying mechanisms of action are. Others and we previously reported that our HBx mice showed hepatomegaly at the age of 8 weeks due to lipid accumulation in hepatocytes and dysplasia with abnormal growth of hepatocytes [51]. HBx mice showed enlarged livers relative to age- matched WT mice even before pathologic phenotype occurred. Our data showed that hapatomegaly in HBx mice was caused by abnormal cell growth, and in the *in vitro* model, HBx causes abnormal cell growth with the appearance of multilayers. Abnormal growth in *in vivo* and *in vitro* models of HBx correlates with expression of fetal liver growth-factor IGF-II.

Previous studies indicated that IGF-II promotes transactivation caused by HBx; and Sp1 phosphorylation induced by HBx is involved in the enhancement of DNA-binding activities of Sp1, which resulted in the up-regulation of IGF-II transcription [52]. Moreover, activated DNMT induced by HBx can cause loss of imprinting. We were able to demonstrate that IGF-II signaling into HepG2-Mock cells through treatment of rhIGF-II or conditioned medium from HepG2-HBx cells enables these cells to promote cell growth. Furthermore, blocking IGF-II signaling with IGF-II-specific neutralizing antibodies could reverse phenotypes, over growth, and especially abnormal multilayer growth. These finding indicate that HBx induced IGF-II might result in blocking cell-to-cell communication through loss of a junction protein such as cadherin and catenin. Normally, hepatocytes adhere to each other through a junctional complex formed by cadherin and catenins. The adult liver expresses E-cadherin and β-catenin abundantly localized at cell–cell junctions to maintain their epithelial character. Unexpectedly, we saw the opposite effect in HBx mice, with epithelial markers such as E-cadherin and α and β-catenin decreasing.

EMT occurring during earlier pathogenesis by HBx does not align with an increase of mesenchymal markers, N-cadherin, or a-SMA. However, during carcinogenesis HBx reduces not only the epithelial markers, E-cadherin and catenin, but also increases the mesenchymal marker N-cadherin.

HBx mice show obvious loss of E-cadherin and the EMT phenotype, however no activation of EMT-related signal pathways, c-Src, Akt, ERK, JNK, p38, STAT3 and NF-kB arose. Also, no differences arose in EMT-inducing transcription factors. Most reports of EMT through loss of E-cadherin were mediated by related transcription factors, whereas other reports suggested posttranslational modifications through MMP, ubiquitin linked pathways. In HBx mice, full length E-cadherin degrades to a truncated form. Suggesting that, at least in part, loss of full length E-cadherin is due to protease. However, compared to loss of E-cadherin, the truncated form was not sufficient enough to explain the result.

Another pathway of proteolysis, is SUMO [27]. Previous studies reported 90 kDa protein detected as the dominant source of SUMO1[53]. In contrast to our expectation, the 90 kDa form of SUMO1 depleted in HBx mice. Evaluation of intact SUMO1 and SUMO2/3 in livers revealed that the free pool of SUMO1 increased in HBx mice. At that time, the conjugated form of SUMO1 decreased. Previous studies revealed that the amount of non-conjugated SUMO2/3 is greater than that of nonconjugated SUMO1, indicating that the SUMO2/3 modification system has a potentially greater capacity to modify cellular proteins than the SUMO1 system [54]. Also SUMO1 monomers can attach to a target molecule. Thus, for SUMO1ylation, SUMO1 needs to change from the conjugated form to the free monomer form.

Based on previous reports and our data, we speculated that monomer SUMO1 increased by dissociation of conjugated SUMO1, which closely related to E-cadherin SUMOylation. As we expected, in the mouse model SUMO1ylated E-cadherin, and to lesser extent, ubiquitinated and SUMO2/3ylated E-cadherin were higher in HBx mice. Analysis of these data suggested that SUMO1ylation of E-cadherin is the dominant mechanism leading to proteolysis of E-cadherin. Increased ubiquitination and SUMO2/3ylation also explained increased SUMO1ylation. SUMO, especially poly-SUMO, can act as a signal for the recruitment of E3 ubiquitin ligases, which lead to the ubiquitylation and degradation of the modified protein [29, 30]. SUMO2/3 together with SUMO1 resulted in poly SUMOylation. Researchers suggested that ubiquitination can activate protein cleavage through recruitment of MMPs. The cleaved form of E-cadherin was detected in the HBx model and can explain SUMO-mediated ubiquitination. Taken together, SUMOylation of E-cadherin may explain the loss of E-cadherin in HBx mice.

Surprisingly E-cadherin degradation through SUMO is different in carcinoma. Consistent with HBx mice, HepG2-HBx cells also showed a decreased E-cadherin protein level but no difference in expression level of mRNA. Also HepG2-HBx cells showed mild ubiquitinated E-cadherin modification. In contrast, in the cancer-cell line, E-cadherin SUMOylation occurred by SUMO2/3 rather than being mediated by SUMO1. Not only SUMOylation but also activated Akt signaling resulted from overexpressed IGF-II, increasing the EMT-inducing transcription factor Twist. Even though HBx induced IGF-II plays a critical role in loss of E-cadherin to lead EMT in early pathogenesis and carcinoma, downstream signal pathways seems more tightly regulated based on environment. Differential regulation of EMT by IGF-II needs to be further investigated.

Here we described previously undescribed mechanisms by which increased IGF-II causes an EMT phenotype, and loss of E-cadherin through a novel mechanism, SUMOylation. These observations suggest that increased levels of IGF-II could serve as potential diagnosis markers for early disease stage.

Also, inhibition of HBx-induced IGF-II might be a therapeutic target for the treatment of liver enlargement and liver cancer.

## MATERIALS AND METHODS

### Transgenic mice

The production of HBx mice used in this study was reported previously [6]. Briefly, HBx homozygous (+/+) mice were produced by mating HBx heterozygous mice with each other. To generate HBx homozygous mice on a mixed background of C57BL/6 and CBA strains, HBx homozygous mice with C57BL/6 background were crossed with CBA WT mice. The heterozygous offspring with a mixed background of C57BL/6 and CBA strains were the cross mated. Among their offspring, HBx homozygous mice were selected by genotyping. Selected mice were then crossed up to F12, which is applicable for the study as an inbred strain with a mixed genetic background (C57BL/6 and CBA). In the current study, these F12 mice were used for *in vivo* analysis. HBx (+/+) mice were verified by polymerase chain-reaction (PCR) analysis. We used PCR primers as follows: one set is the sense primer 5′-ttctcatctgccggtccgtg-3′ and antisense primer 5′-gggtcaatgtccatgcccca-3′ and another set was the sense primer 5′-gaaaacacactcactgttcagag-3′ and antisense primer 5′-gtaagccgctttctcttatgcag-3′. The WT mice were derived from littermates between HBx heterozygous male and female mice, with a mixed genetic background (C57BL/6 and CBA). Mice were housed in a specific pathogen-free environment. All animal procedures were specifically approved by the Institutional Animal Welfare, Care and Use Committee, Korean Research Institute of Bioscience & Biotechnology (KRIBB, Daejeon, Korea) and conducted in accordance with the guidelines.

### Plasmid construction and transfection

We constructed the expression vector, pcDNA3-HA-HBx, as follows. A 475bp HBV subtype *adr* CDS fragment was subcloned into an EcoRI/XhoI site of a pcDNA3-HA tagged vector, and then CMV promoter DNA in the vector was replaced with an HBV X-gene authentic promoter DNA derived from a pHEX1 vector. HepG2 cells were plated in 6-well culture plates for 24 hr prior to transfection. Cells were transfected with 3 μg of pcDNA3-HA-HBx constructs using a Lipofectamine 2000 reagent (Invitrogen, CA, USA) according to the manufacturer's instructions. After 48 hr, cells were trypsinized and plated in a medium containing 1000 μg/ml G418. Following selection for 2 weeks, total populations of G418-resistant cells were pooled and single-cell sorted into 96-well plates with a growth medium containing 1,000 μg/ml G418. Sorted single cells were grown under selection for an additional 2 weeks and expanded into stable cell lines. The candidate clones were analyzed by RT-PCR analysis with a specific HBx primer.

### Cell line and cell-culture conditions

HepG2–HBx cells were grown in an atmosphere containing 5% CO_2_ at 37°C in Dulbecco's modified eagle medium supplemented with 10% fetal bovine serum, 100 units/ml penicillin, and 100 units/ml streptomycin.

### Immunohistochemistry

4-μm-thick sections were cut from the formaldehyde-fixed and paraffin-embedded tissue specimens and mounted on charged glass slides. Samples were incubated overnight at 4 °C with anti-IGF-II rabbit polyclonal antibody (1:200; Santa Cruz Biotechnology, CA, USA). The sections were then incubated with horseradish peroxidase–conjugated anti-rabbit IgG for 30 min at room temperature, and 3,3′-diaminobenzidine(DAB) substrate chromogen solution was applied. Finally, the sections were counterstained with hematoxylin.

### Western blot analysis

Proteins (20 ug/sample) were separated on 12% sodium dodecyl sulfate-polyacrylamide gels and transferred to nitrocellulose membranes (Millipore, Bedford, MA, USA). The membranes were blotted at 4 °C overnight with primary antibodies. The membranes were washed five times with 10-mM Tris–HCl (pH 7.5) plus 150-mM NaCl (Tris buffered saline, TBS) that contained 0.2% Tween-20, and incubated with horseradish peroxidase-conjugated IgG. After the removal of excess antibodies by washing with TTBS, specific binding was detected using a chemiluminescence detection system (Amersham, Berkshire, UK) according to the manufacturer's instructions. Mouse monoclonal Ab to IGF-II, phospho-ERK, SUMO1 and rabbit polyclonal Abs to SUMO2/3 was from Santa Cruz Biotechnology. Rabbit polyclonal Abs to phospho-p65, phospho-src, phospho-STAT3 and mAbs to ubiquitin were purchased from Cell Signaling Technology (Beverly, MA, USA). Mouse monoclonal Ab to E-cadherin was from BD science. Rabbit polyclonal Abs to GAPDH were from Lab Frontier (Seoul, Korea), and horseradish peroxidase conjugated goat Abs to mouse or rabbit IgG were from Amersham and Sigma (Sigma-Aldrich, MO, USA).

### RNA isolation and q-PCR analysis

Total RNA was isolated from the HepG2 cells, or liver tissues from mice, using a TRIzol reagent (Invitrogen, CA, USA) according to the manufacturers' specifications. The concentration of total RNA in the final elutes was determined by nano-drop. Total RNA was converted into single-strand cDNA using a cDNA synthesis kit (Fermentas, Glen Burnie, MD, USA). Amplification of the target genes by real-time RT-PCR was conducted using SYBR Green PCR premix Ex Taq (TAKARA, Shiga, Japan) followed by analysis using the ExicyclerTM 96 Real-Time Quantitative Thermal block (Bioneer, Daejeon, Korea). Relative gene expression was calculated using the comparative Ct (2-δδCt) method. The primers used for real-time PCR are shown in supporting information, Supplementary materials and methods.

### Isolation of primary hepatocytes

We isolated hepatocytes using the methods previously reported [55]. To briefly introduce it here, perfusion was accomplished through the inferior vena cava with EGTA contained HBSS and collagenase (Sigma-Aldrich, MO, USA) contained L-15 media. After collagenase perfusion the liver capsule was disrupted and digested liver parenchyma was suspended with an FBS-added media. Afterward, filtered and washed hepatocytes were isolated through percoll (GE science, WA, USA) gradient methods. For BrdU incorporation analysis (BrdU Flow Cytometry Assay Kit, BD Biosciences, CA, USA), cells were incubated in DMEM (containing 10% FBS) in the presence of 10 mM BrdU at 37°C for 24 hours.

### Co-immunoprecipitation for ubiquitination and sumoylation assay

With/without 20uM of MG132 treatment cell lysate was quantified using the Bio-Rad Protein Assay Kit (Bio-Rad Laboratories, Hercules, CA, USA). Co-immunoprecipitation was performed with a mouse anti-E-cadherin antibody (BD science, CA, USA) Western blot analysis was performed using Mouse anti-ubiquitin (Cell Signaling Technology, Beverly, MA, USA), and anti-sumo1 and rabbit anti-sumo2/3 antibody (Santa Cruz Biotechnology, Santa Cruz, CA, USA).

### Statistical analysis

All data are presented as the mean ± *SD*. A two-tailed Student's *t* test was used to evaluate significance; *p* values < 0.05 were considered statistically significant, and values < 0.01 were considered highly significant.

## SUPPLEMENTARY MATERIALS METHODS AND FIGURES



## Abbreviations

CM: conditioned media
DNMT: DNA Methyltransferases
MDM2: Mouse double minute 2 homolog
MMP: Matrix metalloproteinase
HCC: Hepatocellular carcinoma
HBV: Hepatitis B virus
HBx: HBV-encoded X protein
HepG2-HBx: HBx-expressing HepG2 cells
IGF-II: Insulin-Like Growth Factor II
IGFIR: Insulin-like growth factor 1 receptor
EMT: Epithelial-mesenchymal transition
SUMOs: Small ubiquitin-like modifiers
Ub: Ubiquitination
a-SMA: Alpha-smooth muscle actin
