# Supplementary material for: IGF-II induced by hepatitis B virus X protein regulates EMT via SUMO mediated loss of E-cadherin in mice
Source: Oncotarget. 2016 Jul 29;7(35):56944–57. doi: 10.18632/oncotarget.10922 (PMC5302964; doi:10.18632/oncotarget.10922)
Supplement: Supplementary file 1 [file oncotarget-07-56944-s001.pdf]

# IGF-II induced by hepatitis B virus X protein regulates EMT via SUMO mediated loss of E- cadherin in mice

## MATERIALS AND METHODS

### The primers used for real-time PCR

The primer sequence were: for mouse 5′-caaagacggaggtgtgctttt-3′ and 5′-aaaatgaatggggatggaca-3′ for MEST, 5′-cgtgggaaaggtgattcagt-3′ and 5′-ttcacactggagctgtcgtc-3′ for Zrsr1, 5′-cgggaggacaaccagactaa-3′ and 5′-agtggtgtagccagcgaact-3′ for GNAS, 5′-ggctggccatagactggata-3′ and 5′-caggacatccagagtcagca-3′ for EGF, 5′-ttcccagctggtctatggtc-3′ and 5′-tggtgctgactgcatttctc-3′ for HGF, 5′-caagctgtgtgtctccgaaa-3′ and 5′-tgattcggttcttccaggtc-3′ for IGF-IR, 5′-gaagatcgtgggtgtgaggt-3′ and 5′-cagtccaggggacctttgta-3′ for IGF-IIR, 5′-ggcattgtggatgagtgttg-3′ and 5′-tctcctttgcagcttcgttt-3′ for IGF-I, 5′-gaggacagtggcaaaagctc-3′ and 5′-tcggatgtgcatcttcagag-3′ for Snail, 5′-cgggtcatggctaacgtg-3′ and 5′-cagcttgccatcttggagtc-3′ for Twist, 5′-cctttctcttgccctcactg-3′ and 5′-acagcagccagactcctcat-3′ for Slug, 5′-gagcaccgccgtcatttatc-3′ and 5′-gtgtaactgcacagggagca-3′ for ZEB1.

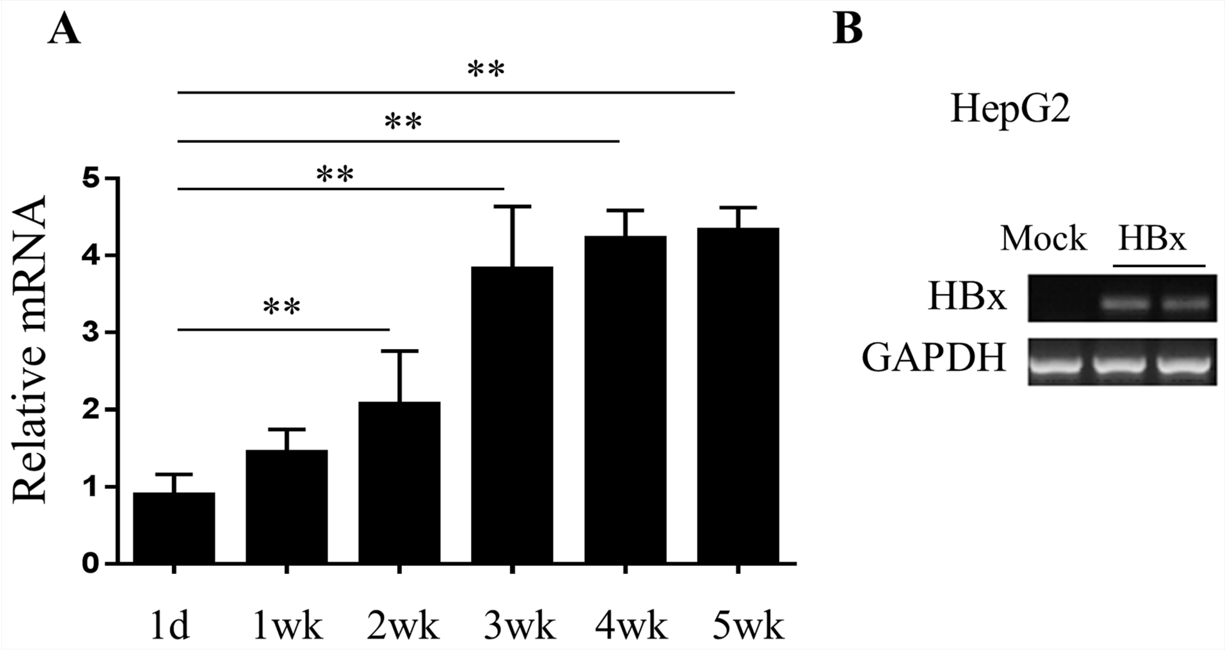


**Supplementary Figure S1: HBx expression in HBx mice and HepG2-HBx cells. A.** HBx expression in HBx mouse livers from day 1 to 5 weeks. **B.** HBx expression in HepG2-Mock and HepG2-HBx cells. Mean ± SD; *n = 9. **p <0.01*

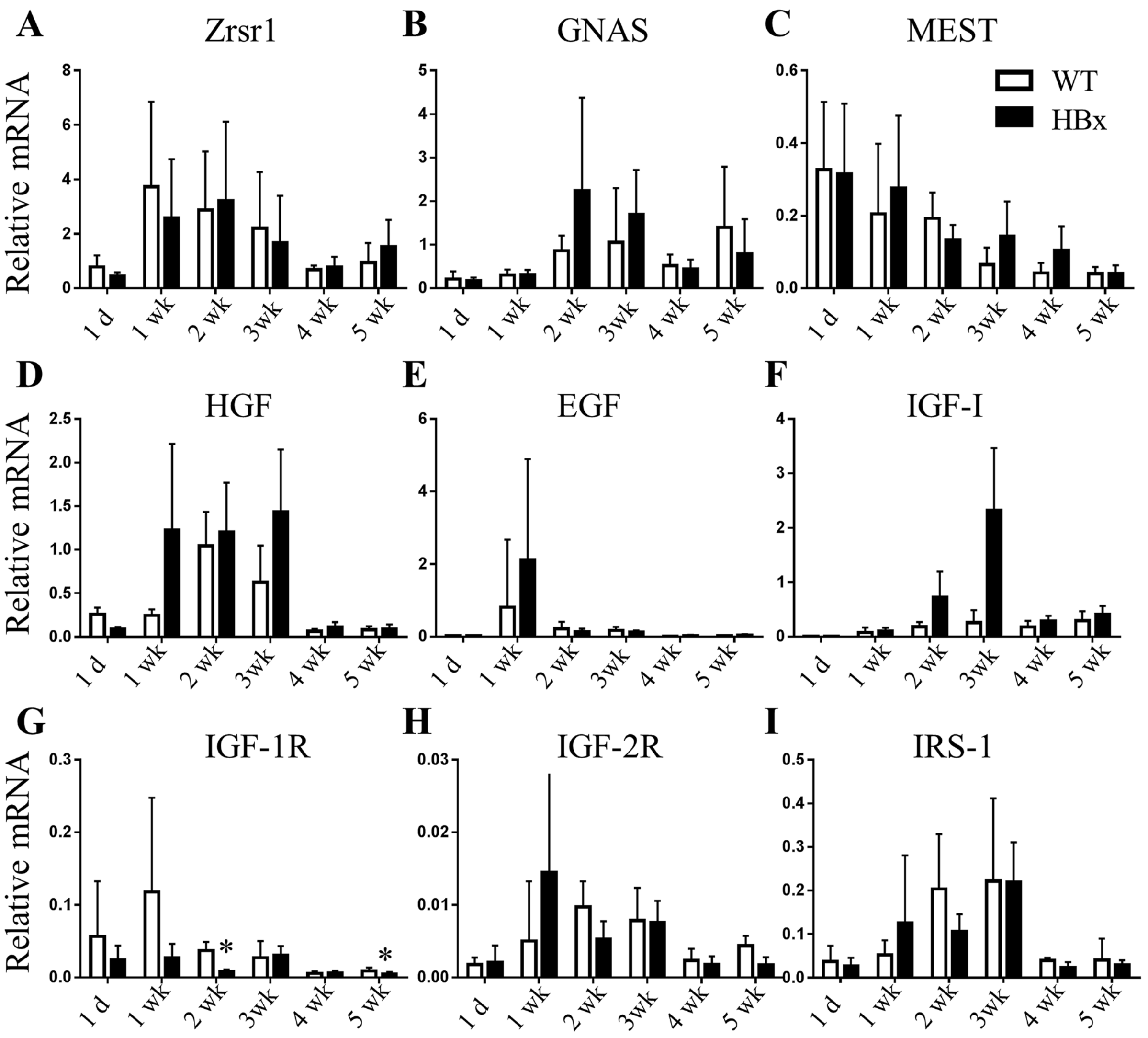


**Supplementary Figure S2: Relative mRNA expression for IGF signaling molecules, growth factors, and imprint genes in the WT and HBx mouse livers from 1 day to 5 weeks.** The expression levels of imprinted genes [Zrsr1 **A.**, GNAS **B.**, MEST **C.**], growth factor genes [HGF **D.**, EGF **E.**], and IGF signaling related genes [ IGF-I **F.**, IGF-IR **G.**, IGF-IIR **H.**, IRS-1 **I.**]. Mean ± *SD*; *n* = 9. **p* <0.05.

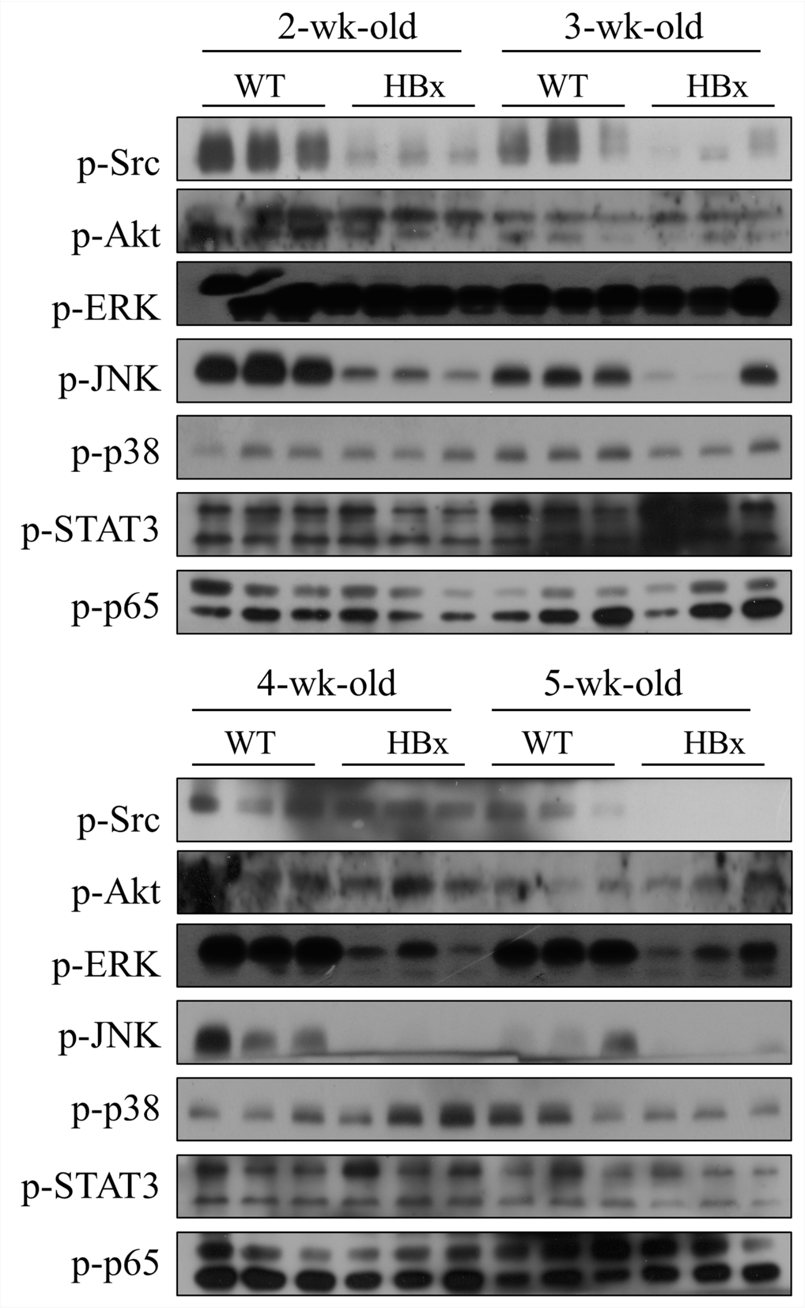


**Supplementary Figure S3: Activation of EMT related signal transduction molecules in mouse livers.** Phosphorylated signaling molecules (c-Src, Akt, ERK, JNK, p38, STAT3 and p65) were detected by western blotting. GAPDH served as loading control.

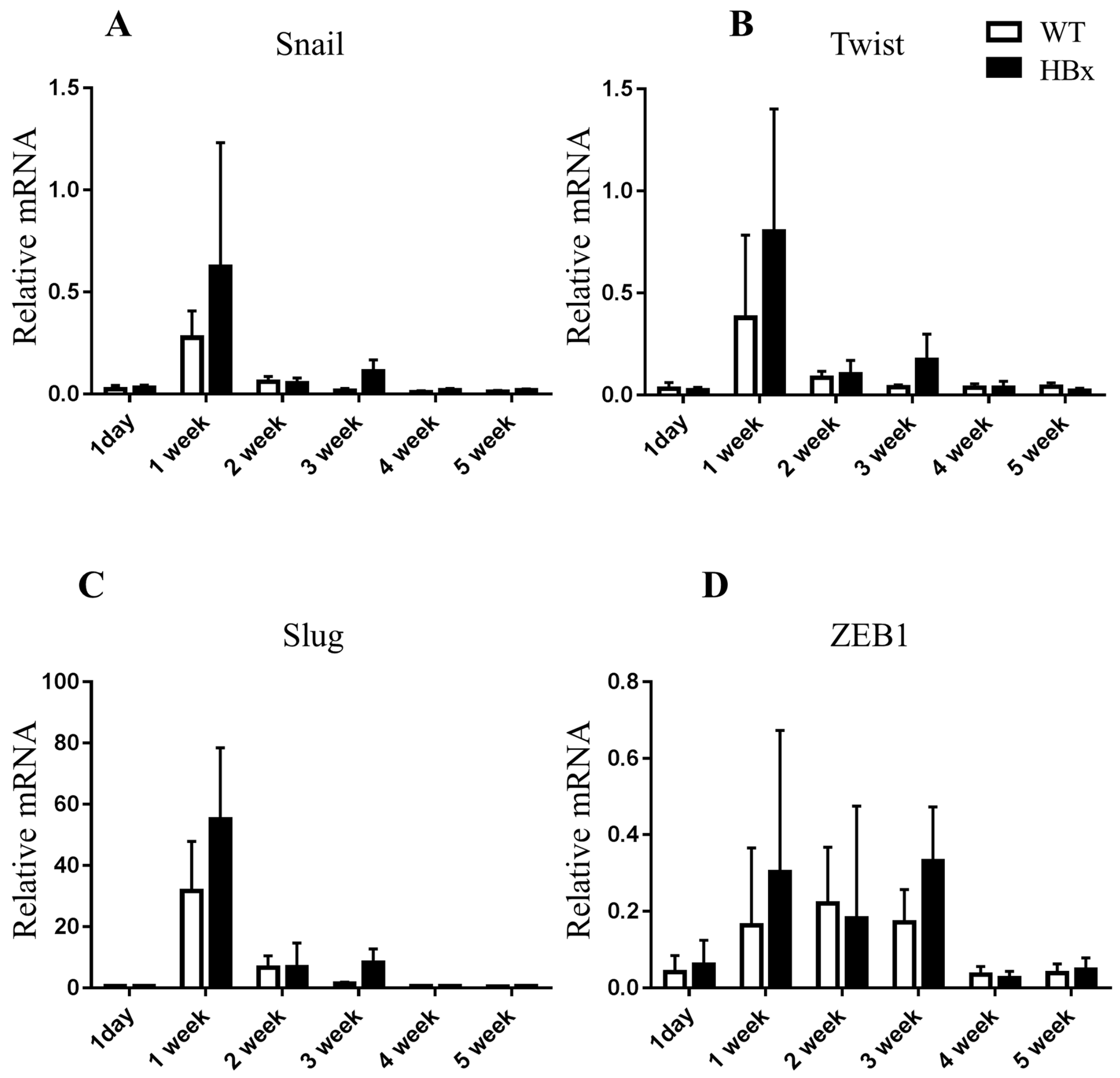


**Supplementary Figure S4: Relative mRNA expression for EMT related transcription factors in the WT and HBx mouse livers from 1 day to 5 weeks.** The gene expression levels of **A.** Snail, **B.** Twist, **C.** Slug and **D.** ZEB1. Mean ± *SD*; *n* = 9.

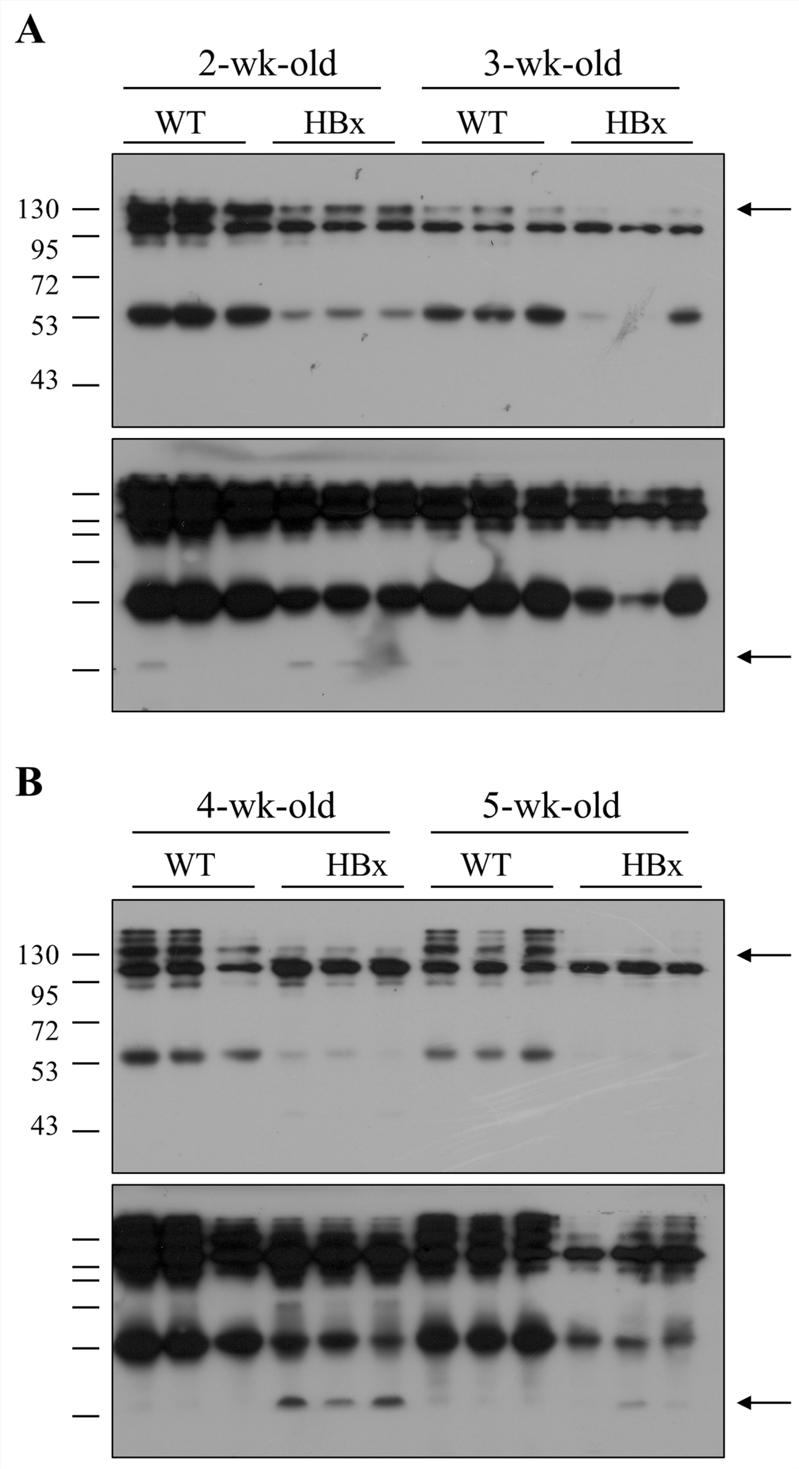


**Supplementary Figure S5: Truncated E-cadherin detected in HBx mice.E-cadherin form detected by western blotting in 2-3weeks. A.** and 4-5weeks **B.** of WT and HBx mouse livers. (Full length of E-cadherin (120 kDa) and truncated intracellular domain (40 kDa) are indicated by arrow. mouse IgG (55kDa).

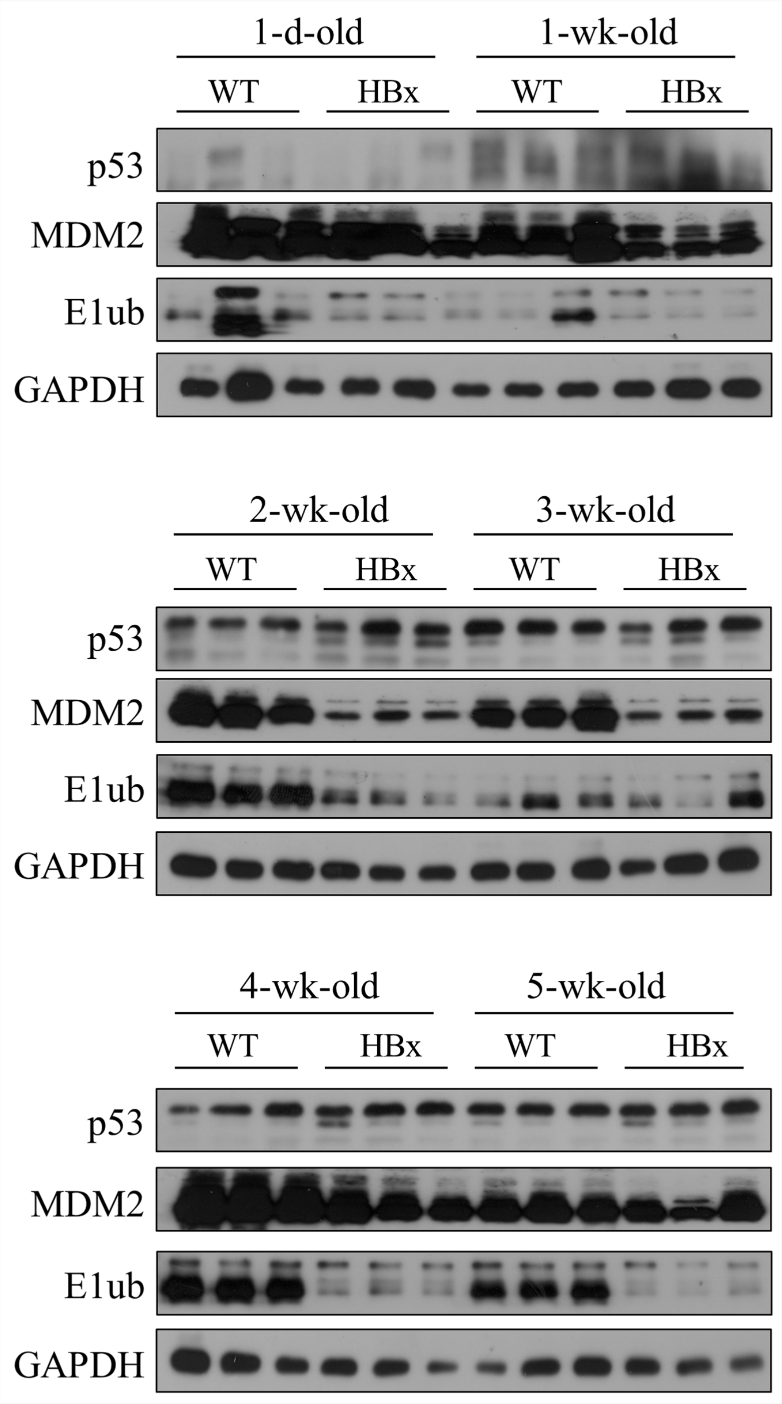


**Supplementary Figure S6: Protein level of ubiquitin E3 enzyme MDM2 in WT and HBx mouse livers.** The expressions of MDM2, its target protein p53 and ubiquitin E1 enzyme were analyzed by western blotting. GAPDH served as loading control.

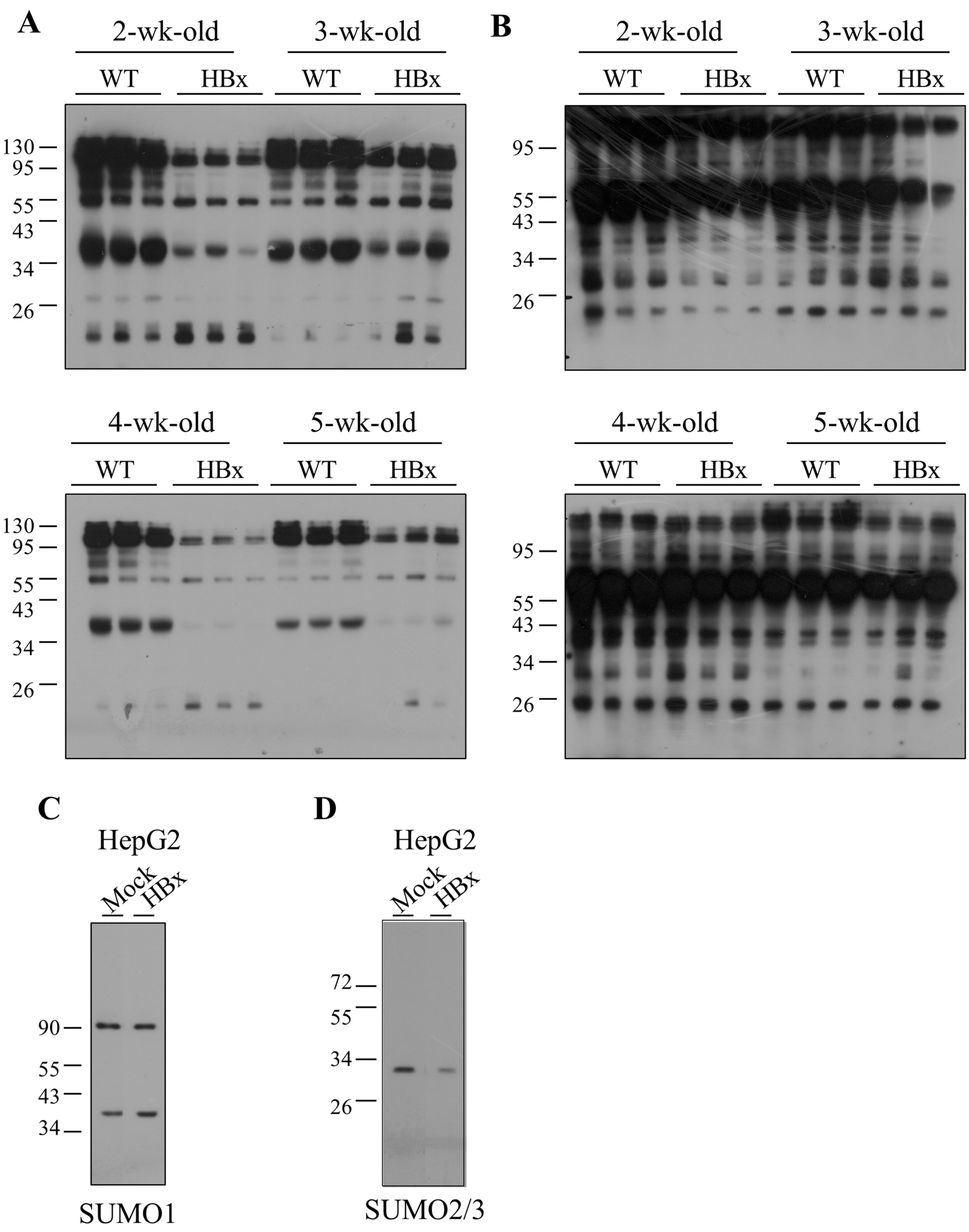


**Supplementary Figure S7: The expression pattern of SUMO in WT and HBx mouse livers and HepG2 cells.** Western blotting was performed to detect SUMO1 **A.** and SUMO2/3 **B.** forms expressed in liver of mice as well as SUMO1 **C.** and SUMO2/3 **D.** protein structure patterns in HepG2-Mock and HBx cells.
